# Supplementary material for: Early lock-in of structured and specialised information flows during neural development
Source: eLife. 2022 Mar 14;11:e74651. doi: 10.7554/eLife.74651 (PMC9064303; doi:10.7554/eLife.74651)
Supplement: Transparent reporting form [file elife-74651-transrepform1.docx]

***eLife’s* transparent reporting form**

We encourage authors to provide detailed information *within their submission* to facilitate the interpretation and replication of experiments. Authors can upload supporting documentation to indicate the use of appropriate reporting guidelines for health-related research (see [EQUATOR Network](http://www.equator-network.org/%20)), life science research (see the [BioSharing Information Resource](https://biosharing.org/)), or the [ARRIVE guidelines](http://www.plosbiology.org/article/info:doi/10.1371/journal.pbio.1000412) for reporting work involving animal research. Where applicable, authors should refer to any relevant reporting standards documents in this form.

If you have any questions, please consult our Journal Policies and/or contact us: [editorial@elifesciences.org](mailto:editorial@elifesciences.org).

**Sample-size estimation**

- You should state whether an appropriate sample size was computed when the study was being designed
- You should state the statistical method of sample size computation and any required assumptions
- If no explicit power analysis was used, you should describe how you decided what sample (replicate) size (number) to use

Please outline where this information can be found within the submission (e.g., sections or figure legends), or explain why this information doesn’t apply to your submission:

As is discussed in section IV A, we are making use of an existing public dataset. As is also discussed in that section, we selected from this dataset the cultures which had multiple (3 or 4) long overnight recordings. These long recordings were necessary for the application of our information-theoretic estimators. This resulted in us choosing four cultures with three to four replicates each.

For the Spearmen correlations calculated in the paper (Figs. 3 - 8), the number of datapoints was determined by the nature of the dataset. We were either using a quantity estimated on each electrode, or one estimated on all pairs of electrodes. As such, the sample size was determined by the number of electrodes. In the cases where we were using all pairs of electrodes, this resulted in a substantial potential sample size of 3422.

**Replicates**

- You should report how often each experiment was performed
- You should include a definition of biological versus technical replication
- The data obtained should be provided and sufficient information should be provided to indicate the number of independent biological and/or technical replicates
- If you encountered any outliers, you should describe how these were handled
- Criteria for exclusion/inclusion of data should be clearly stated
- High-throughput sequence data should be uploaded before submission, with a private link for reviewers provided (these are available from both GEO and ArrayExpress)

Please outline where this information can be found within the submission (e.g., sections or figure legends), or explain why this information doesn’t apply to your submission:

As discussed in the previous answer, we used the data from four separate cultures with three to four replicates per culture. We have computed results separately for each replicate of each culture.

No data was excluded from the statistical analysis. Outliers were handled by the use of Spearman correlations. We excluded outliers from the scatter plots presented in Fig 1. The caption of this figure clearly details how they were removed. These outliers were not excluded from further downstream analysis.

**Statistical reporting**

- Statistical analysis methods should be described and justified
- Raw data should be presented in figures whenever informative to do so (typically when N per group is less than 10)
- For each experiment, you should identify the statistical tests used, exact values of N, definitions of center, methods of multiple test correction, and dispersion and precision measures (e.g., mean, median, SD, SEM, confidence intervals; and, for the major substantive results, a measure of effect size (e.g., Pearson's r, Cohen's d)
- Report exact p-values wherever possible alongside the summary statistics and 95% confidence intervals. These should be reported for all key questions and not only when the p-value is less than 0.05.

Please outline where this information can be found within the submission (e.g., sections or figure legends), or explain why this information doesn’t apply to your submission:

We have plotted a large amount of raw data and intermediate results in the many scatter plots in Figs. 1 and 3-8.

The core value that we are estimating from the data, and performing further analysis on, is the transfer entropy (TE). Section IV D describes in detail how we go about estimating this value. Moreover, we make careful use of surrogate data in order to estimate a null distribution under the hypothesis of zero TE. This allows us to perform significance tests for the TE values being different from zero. This testing procedure is described in section IV F.

(For large datasets, or papers with a very large number of statistical tests, you may upload a single table file with tests, Ns, etc., with reference to sections in the manuscript.)

**Group allocation**

- Indicate how samples were allocated into experimental groups (in the case of clinical studies, please specify allocation to treatment method); if randomization was used, please also state if restricted randomization was applied
- Indicate if masking was used during group allocation, data collection and/or data analysis

Please outline where this information can be found within the submission (e.g., sections or figure legends), or explain why this information doesn’t apply to your submission:

There were no samples collected or experimental groups in this work. As mentioned above, we are using an existing open dataset. All details are available in the publication associated with that dataset (Wagenaar et. al. 2006).

**Additional data files (“source data”)**

- We encourage you to upload relevant additional data files, such as numerical data that are represented as a graph in a figure, or as a summary table
- Where provided, these should be in the most useful format, and they can be uploaded as “Source data” files linked to a main figure or table
- Include model definition files including the full list of parameters used
- Include code used for data analysis (e.g., R, MatLab)
- Avoid stating that data files are “available upon request”

Please indicate the figures or tables for which source data files have been provided:

The *in vitro* data used in this work is part of an existing openly available dataset and can be found at <http://neurodatasharing.bme.gatech.edu/development-data/html/index.html>.

The code used for data analysis can be found here: <https://bitbucket.org/dpshorten/cell_cultures/src/master/>. The script which simulated the developing STDP network (presented in results subsection F) can also be found at this location.
